# Psychometric properties of the Brazilian-adapted version of Sport Imagery Questionnaire

**DOI:** 10.1186/s41155-017-0075-7

**Published:** 2017-10-04

**Authors:** Alberto Filgueiras, Craig R. Hall

**Affiliations:** 1 Departamento de Fundamentos em Psicologia, Universidade do Estado do Rio de Janeiro, Rua São Francisco Xavier, 524, sala 10030, Bloco E, Maracanã, Rio de Janeiro, RJ 20550-900 Brazil; 2 School of Kinesiology, Western University, London, ON Canada

**Keywords:** Imagery, Sport psychology, Performance, Psychometrics, Cross-cultural adaptation

## Abstract

Imagery can be defined as the ability to represent and rehearse in the mind behaviors related to a given situation. The Sport Imagery Questionnaire was developed to measure the frequency of imagery use among athletes. The present study aimed to adapt and to evaluate the psychometric properties of the Brazilian version of this instrument. Study 1 appraised content validity using five sport scientists as judges to quantify the quality of the adaptation for each item; then the Content Validity Coefficient was calculated. Study 2 had 260 athletes from six types of sport answer the Brazilian questionnaire. Exploratory and confirmatory factor analyses were conducted to test factorial validity, Cronbach’s alpha was calculated to assess reliability, and comparisons between groups were used as criterion validity. Study 1 results showed good quality of the adaptation according to the judges. Study 2 showed a 5-factor latent structure which corroborates with the literature. Reliability of the scale was high (*α* = .91), whereas separately subscales ranged between Motivational General: Arousal (*α* = .87) and Motivational Specific (*α* = .94). Regarding group differences, sex showed no significant difference between men and women (*p* = .55; *d* = .09) and neither did levels of practice between amateur, semi-professionals and professional athletes (*p* = .71; *f* = .07). Types of sports revealed moderate effect size and significantly less imagery practice among synchronized swimming, football and beach volleyball athletes, whereas mixed martial artists showed higher frequency of imagery (*p* < .05; *f* = .23). Factor structure, reliability and validity of mixed groups are evidence of a successful cross-cultural adaptation of the Sport Imagery Questionnaire to Brazil.

## Background

Imagery can be defined as the ability to represent and rehearse in the mind behaviors related to a given situation (Filgueiras, 2016a; Rúbio, 2008). Researchers investigated the role of imagery in different situations. For example, there are evidence suggesting that imagery helps to improve working memory (WM) (Turley-Ames, 2003). Language acquisition relies on imagery to boost second language learning among children and adolescents (Dörnyei and Chan, 2013). Even mathematics is able to use imagery as strategy to improve geometry and arithmetic learning among children (Cheng and Mix, 2014).

Imagery is employed extensively in sport as a pivotal intervention conducted by sport psychologists to help improve athlete performance (Hall, Rodgers, and Barr, 1990; Kizildag and Tiryaki, 2012). There is a considerable amount of evidence suggesting that imagery boosts performance through: motivation (Kizildag and Tiryaki, 2012; Lebon, Collet, and Guillot, 2010), motor and technical learning and tactical planning (Filgueiras, 2016a; Slimani, Chamari, Boudhiba, and Chéour, 2016), cognitive dimensions such as WM, attention and emotional self-control (Mesagno and Mullane-grant, 2010). Accordingly, there is evidence that athletes show individual differences in frequency of imagery use, clarity, and control of mental representation (Hall et al., 1990).

The most widely employed theoretical model of imagery was proposed by Paivio (1985) and later modified by Hall, Mack, Paivio, and Hausenblas (1998) which resulted in a five-factor model with two high-order dimensions: cognition and motivation. Cognitive imagery can be divided into Cognitive General (CG) and Cognitive Specific (CS). CG involves imaging competitive tactics, strategies and planning, and CS entails imagery related to the learning and execution of specific technical skills. Motivational imagery consists of Motivational General Mastery and Arousal (MG-M and MG-A, respectively) and Motivational Specific (MS). MG-M imagery is related to controlling and mastering difficult situations, MG-A imagery involves imagery of the excitement, anxiety, and emotional arousal associated with sport, and MS imagery entails winning and achieving goals (Hall et al., 1998; Paivio, 1985; Ruiz and Watt, 2014).

The main instrument used to assess the frequency of imagery use among athletes is the Sport Imagery Questionnaire (SIQ) (Hall et al., 1998). Based on Paivio’s model, the SIQ is a 30-item questionnaire with a Likert-type scale of seven categories of endorsement, ranging from 1 “never/rarely” to 7 “often.” This measure has six items for each one of the five factors of Paivio’s model, with questions such as: “I can consistently control the image of physical skill” for CS, “I make up new strategies in my head” for CG, “I image myself to be focused during a challenging situation” for MG-M, “I image myself handling the stress and excitement of competitions and remaining calm” for MG-A, and “I image myself winning a medal” for MS. Research employing the SIQ has shown that performance and motivation are linked to the frequency of imagery use (Gregg, Hall, McGowan, and Hall, 2011; Issac, 1983; Ruiz and Watt, 2014), thus it seems rather important to assess precisely and reliably these psychological dimensions.

### Cross-cultural adaptation and validity of SIQ

Since Rio de Janeiro was chosen the home of 2016 Summer Olympics, investments in sport psychology have been made by the Brazilian Olympic Committee to ensure good results of Brazilian Olympians (Ministry of Sports of Brazil, 2014). A major part of any intervention program is the assessment of its results. Despite of having sport psychologists employing imagery with athletes, Brazil does not have an adapted and validated measure to assess imagery use (Federal Psychological Council, 2016). This means that, according to the Brazilian Federal Psychological Council (CFP), its professionals are unable to assess quantitatively the impact of what they are doing in the minds of Brazilian athletes regarding imagery, because they lack the necessary measurement instruments. In a country that hosted the last Olympic Games and plays a pivotal role in the world of sports, the importance of having SIQ adapted and validated in Brazilian is unquestionable. On the other hand, Brazil exports athletes to the whole world, such as football, basketball, volleyball and tennis players, swimmers, golfers, cyclers, among other sports. It means that sport psychologists and sport scientists in the whole world would benefit to have a Brazilian Portuguese version of SIQ to use with Brazilian athletes.

In fact, there is a Portuguese-version of SIQ translated to Portuguese; however, Portugal’s Portuguese is quite different from Brazil’s Portuguese which impairs its use among Brazilian athletes. Also, the Portuguese study is exploratory and it does not provide psychometric properties of the measure (Ribeiro et al., 2015). Although those two forms of language seem very similar, evidence suggests that lexical (Barreiro, Wittmann, and de Jesus Pereira, 1996), semantic (de Baubeta, 1992), phonological and pragmatic differences (Fernandes, 2004) are quite significant, which justifies the development of a Brazilian specifically adapted version.

A SIQ Brazilian-translated version was produced, but cross-cultural adaptation procedures are no reported and no psychometric properties were examined (Filgueiras, 2016b). Accordingly, SIQ is already adapted and its psychometric properties studied in three other languages: Finnish, Turkish and Spanish (there is also a Russian version that still requires psychometric analysis). The Finnish-adapted version of SIQ was published in 2006 (Watt, Jaakola, & Morris, 2006). It confirmed (CFA) five factors as hypothesized by Paivio’s model and demonstrated by the original SIQ. The internal consistency as measured by Cronbach’s alpha ranged from 0.64 (CG) to 0.83 (MS). The Turkish version of SIQ was published in 2011 (Kafkas, 2011). A three-factor solution was found using exploratory factor analysis and these dimensions were termed: Cognitive General (CG), Motivational and Cognitive Specific (MGS) and Motivational General-Mastery (MG-M), which means that items from three factors in the original scale (MG-A, MS and CS) were grouped into a single factor, MGS. Internal consistency using alpha ranged from 0.66 (MGS) to 0.87 (MG-M). Kafkas (2011) also conducted a test-retest study of 15 days, showing a correlation of 0.60 (MGS), 0.68 (CG), 0.86 (MG-M) and 0.85 for total score. Correlation between factors ranged from 0.43 to 0.79. Regardless, there is another Turkish-adapted version of SIQ published 1 year after Kafkas (2011) article that shows different results (Vurgun, Dorak, and Ozsaker, 2012). This second Turkish version presents better internal consistency as measured by Cronbach’s alpha ranging from 0.83 (MG-A) to 0.91 (MS). In the exploratory factor analysis (EFA), a five-factor solution was found as well. The Russian version was also created in 2011 (Veraska and Gorovaya, 2011) and while its psychometric properties are yet to be studied, it showed good construct validity by correlating moderately-to-strongly (ranging from 0.63 to 0.75) with the figurative form of Torrance Test of Creative Thinking.

Finally, a Spanish version of the SIQ (Ruiz and Watt, 2014) demonstrated a five-factor solution in both exploratory and confirmatory factor analyses. The internal consistency using Cronbach’s alpha showed good results ranging from 0.72 to 0.86. Correlation between factors ranged from 0.40 to 0.70. A fascinating finding was also reported in this study. Ruiz and Watt (2014) conducted a MANOVA considering sports classification as an independent variable (8 levels: athletics, combat sports, aquatics, transition sports, cycling, invasion contact ballgames, invasion non-contact ballgames, and non-invasion ballgames) and SIQ’s five subscales as dependent variables. Combat sports athletes tended to use significantly more CG imagery than aquatics and non-invasion ballgames athletes and they employed more CS imagery than athletics, aquatics, and invasion non-contact ballgames athletes. Also, athletes from invasion contact ballgames reported significantly higher frequency of MS imagery use when compared to cyclists. These findings taken together point to a possible relationship between sport category and the use of different types of imagery.

Extending previous research adapting the SIQ to various languages, the purpose of this study was to develop the Brazilian cross-culturally adapted version of the SIQ and assess its psychometric properties in a Brazilian sample of athletes. To accomplish this endeavor, two studies were conducted. First, a content analysis using the Content Validity Coefficient (CVC) to assess items’ quality of adaptation according to five independent judges was undertaken (Filgueiras et al., 2015). Second, a psychometric properties investigation with three analyses was conducted: exploratory and confirmatory factor analyses using a sample split-half technique (Hair, Black, Babin, and Anderson, 2010) to assess factorial validity, a reliability study using Cronbach’s alpha such as used in other SIQ studies (Cohen and Swerdlik, 2009; Cronbach, 1951; Hall et al., 1998; Kafkas, 2011; Ruiz and Watt, 2014; Watt et al., 2006), and a comparison between sports categories using null-hypothesis tests similar to Ruiz and Watt (2014) that could produce evidence for validity of mixed groups.

## Methods

### Study 1

#### Participants

Researchers and college faculty were invited to form a panel of specialists in sport psychology to assess the quality of adaptation of the Brazilian-version of SIQ (Filgueiras, 2016b). Among researchers who accepted the invitation (*n* = 5), three fields of the sports sciences were represented: sport psychology (*n* = 3), physiology (*n* = 1), and physical education (*n* = 1).

#### Procedure

Procedures for translation and adaptation followed the guidelines of the ITC (2010). First, the authorization to translate was obtained with the author of the original instrument and the publisher. Three professional bilingual translators whose first language (L1) was Brazilian Portuguese translated independently. A synthesis of the three translated versions was generated by the first author (AF) and his research team. Adaptations for the Brazilian culture were included in this step. The translation was,then published (Filgueiras, 2016b), and other Brazilian researchers were invited to contribute via e-mail with new suggestions. The synthesis of this work was presented to five judges who were asked to assess the quality of adaptation using a Likert-type scale ranging from 1 “very poor” to 5 “very good” in each of the 30 items of SIQ, the instructions and categories of response. Items that obtained poor statistical results were modified accordingly by the first author and his students involved in the project. A back-translated version of the instrument made by a professional bilingual translator whose native language was English was sent to the author of the original SIQ (ITC, 2010). His suggestions were incorporated to generate the final version of SIQ:Brazil (SIQ:BR). The present study was approved by the Rio de Janeiro State University Ethical Committee under protocol #180/2016.

#### Data analysis

Participants’ answers were gathered and the content validity coefficient (CVC) was used to assess quality of adaptation (Filgueiras et al., 2015). The CVC algorithm was developed to evaluate pattern of responses and level of agreement between judges and it followed four simple steps to generate coefficients for items, categories of response, instructions, and the scale as a whole. The first step was to calculate the item’s CVC (CVCi). The answers of the participants were then divided by the maximum value of the scale, which corresponded to values between 0.2 and 1.0. The mean of participants’ division was the CVCi. The second step was the coefficient for participants (CVCp); it was used to assess the validity content of the scale as a whole. All answers of each specialist were summed and divided by the maximum sum possible. Thirdly, participants’ polarization (Pp) was generated by dividing the integer 1 by the number of specialists, and then elevating it to the number of participants; because the present study had five participants, the Pp was .00032. Finally, the fourth step was to calculate the test’s CVC (CVCt) that was given by the averaged of the subtraction of Pp from the CVCp. The literature recommends both CVCi and CVCt show values above .80 (Filgueiras et al., 2015). All analyses were conducted in the Microsoft Office Excel.

#### Results and discussion

Overall, content validity of the Brazilian-adapted version of SIQ was good. Some items had to be adapted in order to be understandable in Brazilian Portuguese or Brazilian sports culture. In item 9, the term “fast vs. slow” was altered to “transitions” because the game pace is not addressed in Brazilian Portuguese in terms of fast or slow, but in terms of presence and absence. In items 12 and 30, the term “successfully” was changed to “well” in the synthesis-version, but returned to the original term after the author of the original SIQ evaluated those items in the back-translation; thus, it has not affected the scale’s original content and sounds only a little awkward, but is still adequate in Brazilian Portuguese.

In items 13, 15, 22, and 24, the term “excited” was adapted to “joy”, “happiness,” or “enthusiasm” depending on the item. The authors of this study understand that excitement is an emotional state associated with the eagerness to play the day of a sport event; however, in Brazilian Portuguese, it represents primarily sexual arousal, which is clearly what the scale does not assesses. The solution was to adapt the translation as adequately as possible by looking each item specifically. Finally, item 26 had a small change; the term “mentally tough” was replaced by “mentally strong”, because “tough” in Brazilian Portuguese also brings the idea of rigidness. On the other hand, “strong” relates to the exact content whenever this construct is addressed in Brazilian sports culture.

Regarding content validity, judges evaluated the whole group of items regarding its quality of adaptation with a CVCt = .88. Among items, only two (items 9 and 13) presented CVCi below .80 as recommended in the literature. Table 1 provides CVCi and CVCt of the SIQ:BR.

**Table 1:** Content validity coefficient of items according to judges scores for adequacy of adaptation and content validity coefficient of the test as a whole

Item (according to its original content)	CVCi adequacy of adaptation
Cognitive specific 1	.80
Cognitive specific 2	.85
Cognitive specific 3	.90
Cognitive specific 4	.90
Cognitive specific 5	.80
Cognitive specific 6	.85
Cognitive general 1	.90
Cognitive general 2	.95
Cognitive general 3	.65*
Cognitive general 4	.90
Cognitive general 5	.85
Cognitive general 6	.80
Motivational specific 1	.75*
Motivational specific 2	.95
Motivational specific 3	.90
Motivational specific 4	.85
Motivational specific 5	.90
Motivational specific 6	.90
Motivational general - arousal 1	.85
Motivational general - arousal 2	.90
Motivational general - arousal 3	.90
Motivational general - arousal 4	.85
Motivational general - arousal 5	.80
Motivational general - arousal 6	.90
Motivational general - mastery 1	.85
Motivational general - mastery 2	1.00
Motivational general - mastery 3	.85
Motivational general - mastery 4	.85
Motivational general - mastery 5	.90
Motivational general - mastery 6	.95
Test’s Content Validity Coefficient (CVCt)	.88

*CVC below criteria

Overall, the results from Study 1 suggest that SIQ was adequately adapted to Brazilian cultural context with the exception of two items: 9. Cognitive General 3 (CG3) “I image each section of an event/game (e.g., offense vs. defense, fast vs. slow)” and 13. Motivational Specific 1 (MS1) “I image the atmosphere of winning a championship (e.g., the excitement that follows winning a championship)”. According to Filgueiras et al. (2015), the CVCi entails a simple statistical index to represent the evaluation of a group of judges regarding a single item and the scale as a whole; the adopted criteria in the literature is equal to and above .80. This means the judges in Study 1 did not agree regarding the quality of adaptation of these two items. The first problematic item CG3 was altered to be adapt to Brazilian culture; the term “fast vs. slow” became “transitions”. Clearly the judges did not agree that this was the best adaptation option. The same phenomenon seemed to happen with item MS1; during adaptation, the word “excitement” was replaced by a few synonyms in Brazilian Portuguese because the literal translation would bring confusion to test-takers due to the sexual connotation of this term in Brazil. Apparently, judges considered that the specific adaptation of this word to “joy” in this item was not keeping the original intent. In fact, there is evidence that “excitement” is quite specific in terms of semantics whenever referred to sports; pre-competitive anxiety is frequently interpreted as exciting and it is not necessarily something good, in fact there is evidence that athletes tend to refer to anxiety as a form of excitement, which does not hold a positive affect content (Brooks, 2014). However, in Brazilian Portuguese, there is no adequate translation; thus, it seems that this phenomenon needs further care in a deeper statistical analysis. Nevertheless, the results from Study 2 can shed some light over the adequacy of SIQ items in Brazilian Portuguese.

### Study 2

Given that Study 1 demonstrated that the quality of the adaptation for the items of the SIQ:BR was adequate, psychometric analyses of the adapted questionnaire was undertaken in the present study.

#### Participants

Elite and amateur athletes were invited to volunteer. They were recruited by the authors of this study in universities, gyms, and competitions. Among 300 athletes invited, 260 (86.7% of adhesion) agreed to participate. The only exclusion criterion was previous psychiatric disorder and no participant declared such condition. Athletes who volunteered were from six sport categories: basketball (*n* = 31), football (*n* = 90), gymnastics (*n* = 59), combat sports (*n* = 22), synchronized swimming (*n* = 16), and beach volleyball (*n* = 42). Average age of the participants was 33.62 (*SD* = 7.68) years-old, ranging from 18 to 48 years of age. Among volunteers, three levels of sport were self-reported: professionals (*n* = 172), semi-professionals (*n* = 36), and amateurs (*n* = 52), but there was no synchronized swimming professional athlete, no amateur beach volleyball player, and all semi-professionals were found among football players. Regarding sex, there were more men (*n* = 191) than women (*n* = 69) in the sample; however, there were sports categories specific per sex, such as football (only men) and gymnastics (only women). Finally, the average time in the same sport in years was 6.00 (*SD* = 4.71), ranging from 3 months to 20 years of engagement.

#### Procedure

One of the authors (AF) and his group of students went to the athletes’ practice facilities such as clubs, gyms, colleges, and schools to invite athletes to participate. Interested participants were provided with a hyperlink to the survey website specifically designed for the present study via e-mail, cell phone message, or paper note. The first page of the survey was the consent form; if the volunteer did not provide consent, then he/she was not being able to proceed to the next page. To make sure the participants remained anonymous, no personal data other than demographics were collected. Next, demographic information was collected: age, sex, sport category, self-reported skill level, time in his/her sport category in months and years, and number of hours of practice per day. Then, participants completed the SIQ:BR. The present study was approved by the Rio de Janeiro State University Ethical Committee.

#### Statistical analyses

First, descriptive statistics, the average, standard deviation (SD), and standard error of the mean (SEM) of the whole scale were calculated considering demographics as independent variables: sex, sport category and skill level. Homogeneity tests, Kolmogorov-Smirnov, were conducted to ensure normal distribution of sample’s data. An independent sample *t* test was conducted to compare imagery use by sex, and a one-way ANOVA was used to examine the results between sport categories and skill levels.

Second, factorial structure was tested trying to find the best possible model based on the evidence in the literature. Two possible solutions were expected; a three factor solution (Kafkas, 2011) merging MG-A, MS, and CS in a single factor or a five factor solution (Hall et al., 1998; Ruiz and Watt, 2014; Watt et al., 2006). The first step was to randomly divide the sample. Numbers were randomly given to each participant when they filled the online survey and odd and even numbers were separated in two files. One was used to conduct the exploratory factor analysis (EFA) and the other to run the confirmatory factor analysis (CFA), according to the split-half technique suggested by the statistical literature (Cohen and Swerdlik, 2009; Hair et al., 2010). The EFA was conducted using the polychoric correlation matrix with the Hull Method for dimensional retention, unweighted least squares (ULS) for factor extraction, and Promin rotation. Those decisions were based on the recommendation for scales such as SIQ: Likert-type scale, thus ordinal data, with the same probability of endorsement in every level of response, no missing data, likelihood of moderate-to-high correlation between factors and good sample size which corresponds to probable parameterized distribution (Jöreskog and Moustaki, 2001; Lorenzo-Seva and Ferrando, 2006; Stucky et al., 2011). In the same extent, factor loadings were considered relevant for values above .40 as recommended by Hair et al. (2010) and Stucky et al. (2011). The CFA was carried out by testing the extracted model from the EFA with the other half of the sample. Since two other models were found by SIQ researchers, the 3- and the 5-factor solutions were tested. The estimation method was maximum likelihood using the covariance matrix according to the suggestions of Hair et al. (2010). Fit indexes considered were: (a) error (root mean square error approximation – RMSEA), (b) index to compare the tested against the null-hypothesis and the independent hypothesis (Comparative Fit Index – CFI), (c) fit index to assess the level of improvement or increment between the tested model and the null-hypothesis (NFI) and (d) index for the information theory goodness of fit which relates to the model parsimony penalizing complex models and prioritizing simpler ones (Bayesian Information Criterion – BIC). The adopted criteria for CFA was RMSEA ≤ .08 (Hair et al., 2010), CFI > .93, NFI > .90 (Byrne, 1994), and the lowest BIC among tested models (A. E. Raftery, 1995). To assess reliability, Cronbach’s alpha (Cronbach, 1951) was calculated for each factor and the scale as a whole.

Finally, a one-way ANOVA were performed using SIQ:BR’s factor-scores separately as dependent variables (CS, CG, MG-A, MG-M, MS) and sport categories as independent variables. Sex was not included in this analysis because the sample was biased by the absence of women in football and men in gymnastics, as well as skill levels due to the lack of professional Synchronized Swimming and amateur Beach Volleyball players; also, semi-professionals were only found among football players. A Bonferroni post-hoc analysis was carried out to conduct pair-to-pair comparisons. Both ANOVA and t-test were confirmed by effect-size using Cohen’s *d* (*t* test) and *f* (ANOVA). Statistical criteria were as recommended by Cohen and Swerdlik (2009): regarding Cohen’s *d*, above .20 is considered a small effect size, above .50 medium and above .80 large; the same categories are adopted to Cohen’s *f* with different values: above .10 small, above .25 medium, and above .40 large.

Descriptive statistics, t-test one-way ANOVA and Cronbach’s alpha were calculated using the package “psych” in the free-software R. Effect size was performed in G*Power free-software. EFA was conducted in the software Factor version 10.4.01 (Lorenzo-Seva and Ferrando, 2006). CFA was carried out in AMOS from the software SPSS 21.0.

## Results

Descriptive statistics, comparisons and effect sizes of the SIQ:BR total score between levels of independent variables are given in Table 2. Kolmogorov-Smirnov tests showed no-significant differences (*p* < .05) across groups: sex, sport categories and level of practice which entails normal distributions of all groups. Because sport category showed statistical differences and small effect size (*d* = .23) between groups, a Bonferroni post-hoc was calculated. It showed that combat sports use imagery significantly more frequently than beach volleyball (*p* = .04), football (*p* = .02), and synchronized swimming (*p* = .05); no other differences were found.

**Table 2:** Mean, standard deviation (SD), standard error of the mean (SEM), and inferential statistics according to independent variables: sex, sport categories, and level of practice

Independent variable	Mean	SD	SEM	Comparison		
				Statistics	*p* value	Effect size
Sex						
Men (*n* = 191)	136.54	32.30	2.34	*t* (258) = −0.60	.55	*d* = .09
Women (*n* = 69)	139.07	28.93	3.48			
Sport Category						
Basketball (*n* = 31)	137.71	33.17	5.96	*F* (5254) = 2.52	.03*	*f* = .23
Football (*n* = 90)	134.14	28.18	2.81			
Gymnastics (*n* = 59)	139.24	28.10	3.66			
Combat sports (*n* = 22)	157.45	35.25	7.52			
Synchronized swimming (*n* = 16)	126.11	29.53	9.84			
Beach volleyball (*n* = 42)	132.77	37.29	5.97			
Level of sport practice						
Professional/elite (*n* = 172)	138.71	25.15	1.92	*F* (2257) = 0.34	.71	*f* = .07
Semi-professional (*n* = 36)	137.56	35.24	5.87			
Amateur (*n* = 52)	133.36	17.03	2.36			

*Significant differences for *p* < .05

The EFA produced a 5-factor solution such as suggested in the majority of the SIQ literature. The polychoric correlation matrix was adequate to conduct the EFA as shown by Bartlett’s statistic (17,911.6; *p* < .001) and the Kaiser-Meyer-Olkin (KMO) test (*KMO* = .87). Table 3 shows factor loadings and proportion of explained variance in percentage. Factor loadings below criterion of .40 were suppressed. Dimensions correlated significantly (*p* < .05) moderate-to-high. The lower correlation was between MG-A and CS (*r* = .22) and the higher correlation was between CG and CS (*r* = .64). Table 4 shows the correlations between factors.

**Table 3:** Factor Loadings of SIQ items according to appearance order and percentage of variance explained by each factor

Item number (in order of appearance in the original scale)	Factor loading				
	Factor 1	Factor 2	Factor 3	Factor 4	Factor 5
Cognitive specific 1			.96		
Cognitive specific 2			.96		
Cognitive specific 3			.86		
Cognitive specific 4			.93		
Cognitive specific 5			.97		
Cognitive specific 6			.88		
Cognitive general 1		.97			
Cognitive general 2		.61			
Cognitive general 3		.96			
Cognitive general 4		.97			
Cognitive general 5		.99			
Cognitive general 6		.99			
Motivational specific 1	.82				
Motivational specific 2	.99				
Motivational specific 3	.98				
Motivational specific 4	.84				
Motivational specific 5	.76				
Motivational specific 6	.86				
Motivational general - arousal 1				.93	
Motivational general - arousal 2				.97	
Motivational general - arousal 3				.68	
Motivational general - arousal 4				.75	
Motivational general - arousal 5				.58	
Motivational general - arousal 6				.54	
Motivational general - mastery 1					.95
Motivational general - mastery 2					.55
Motivational general - mastery 3					.57
Motivational general - mastery 4					.67
Motivational general - mastery 5					.45
Motivational general - mastery 6					.98
Percentage of explained variance	44.79%	17.19%	14.97%	10.39%	5.50%

Factor loadings above .40 were suppressed

**Table 4:** Pearson correlation matrix of SIQ factors

Factor	Correlation			
	CS	MG-A	MG-M	MS
CG	.64	.30	.32	.28
CS		.22	.35	.49
MG-A			.27	.24
MG-M				.50

All correlations were significant *p* < .05

Reliability measured by Cronbach’s alpha revealed adequate consistency of the SIQ:BR. The overall scale’s alpha was high (*α* = .91); CS (*α* = .93), CG (*α* = .89), MG-M (*α* = .90), MG-A (*α* = .87), and MS (*α* = .94). All alphas were above the classic criteria (*α* ≥ .70) (Cohen and Swerdlik, 2009; Cronbach, 1951).

Because results from the EFA showed five dimensions, the authors choose to test only the two models suggested in the literatures in the CFA. The 5-factor solution showed good fit indexes, but the structure of three dimensions were relatively poor. Table 5 provides the fit indexes for the two models.

**Table 5:** Fit indexes for the 3- and 5-factor models tested using CFA

Fit index	Model	
	3 factors	5 factors
*RMSEA*	.11	.07
*CFI*	.76	.94
*NFI*	.63	.89
*BIC*	4765.36	2708.90

Finally, ANOVA results showed significant differences with small effect size (i.e., Cohen’s *f* between .10 and .25) in three of the five types of imagery (CG, MS, and MG-A). These results are shown in Table 6. No significant findings emerged for CS and MG-M imagery.

**Table 6:** Mean and standard deviation (SD) of the six items in each SIQ subscale by sport categories

	Mean (*SD*)				
	CS	CG	MG-A	MG-M	MS
Sport categories					
Basketball (*n* = 31)	5.01 (1.12)	4.89 (1.42)	4.78 (1.23)	5.03 (1.19)	4.94 (1.14)
Football (*n* = 90)	5.03 (1.19)	4.87 (1.22)	4.94 (1.09)	4.98 (1.03)	4.68 (1.30)
Gymnastics (*n* = 59)	4.93 (1.34)	4.34 (1.33)	4.29 (1.45)	4.87 (1.26)	4.71 (1.05)
Combat sports (*n* = 22)	4.87 (1.56)	4.93 (1.71)	5.21 (1.54)	5.10 (1.01)	5.19 (0.87)
Synchronized swimming (*n* = 16)	5.02 (1.87)	4.94 (1.32)	4.97 (1.34)	4.71 (0.93)	4.84 (1.40)
Beach volleyball (*n* = 42)	4.99 (1.25)	5.13 (1.03)	4.75 (0.97)	5.11 (1.05)	4.99 (1.08)
One-way ANOVA	*Statistics*				
F (degree of freedom)	F (5254)	F (5254)	F (5254)	F (5254)	F (5254)
Statistics	0.94	3.03	4.25	1.66	2.48
*p* value	.45	.01	< .01	.14	.03
Effect-size (Cohen’s *f*)	.04	.18	.19	.07	.11

Those are not mean of the sum of items scores in each subscale, but the mean of each person’s average in each subscale. Sum of subscales range from 6 to 42 for one person, whereas average of items range from 1 to 7 for one person

Post-hoc analyses revealed differences in CG, MS and MG-A, it means CS and MG-M did not show any difference between sport categories. Beach volleyball had higher factor-scores in CG than gymnastics (*p* = .045); combat sports presented higher MS scores than football (*p* = .012), gymnastics (*p* = .023), and basketball (*p* = .026), also higher MG-A than beach volleyball (*p* < .001).

## Discussion

The aim of the present study was to determine the psychometric properties of SIQ:BR in a sample of amateur and professional athletes. Initial analyses indicated that there was no significant difference between males and females for the SIQ:BR’s total score. Indeed there was no expectation of finding any difference because previous psychometric (Ruiz and Watt, 2014; Watt et al., 2006) and empirical studies (Kizildag and Tiryaki, 2012) also shown no differences between males and females in their use of imagery. Regardless, literature still lacks solid evidence suggesting that men and women actually do not differ nonetheless; both genders were combined in the factor analyses.

Factor analyses supported previous studies suggesting a 5-factor latent structure of the SIQ (Hall et al., 1998; Ruiz and Watt, 2014; Watt et al., 2006). It is interesting that latent structure of SIQ remains the same regardless of country it was adapted for; Finland (Watt et al., 2006), Spain (Ruiz and Watt, 2014) and, now, Brazil. Also, previous studies in the original language (i.e., English) showed the same results (Hall et al., 1998). The only exception was the Turkish-adapted version of SIQ that showed a 3-factor solution (Kafkas, 2011), probably due to aspects of this specific adaptation rather than a difference in the assessed construct itself. Indeed, Vurgun et al. (2012) contributed with a different Turkish-adapted version of SIQ and a 5-factor solution was found, which strengthen the hypothesis that Kafkas (2011) version had a 3-factor structure due to particularities of this specific adaptation. Accordingly, Paivio’s model (Paivio, 1985) is able to show good dimensionality invariance through cultures from North America (Hall et al., 1998), to Europe (Ruiz and Watt, 2014; Watt et al., 2006) and, now, South America.

Another important aspect of the present results was the low-to-moderate correlations between factors, whereas previous studies diverge on this point. For example, Kafkas (2011) in the Turkish adaptation, as well as Ruiz and Watt (2014) in the Spanish version found moderate-to-high correlations between dimensions, whereas SIQ:BR showed a range between CS and MG-A (*r* = .22) and CS and CG (*r* = .64). The results of the present study are in line with both the correlations reported between factors in the original SIQ (Hall et al., 1998). Thus, it seems as though the SIQ:BR adequately assesses the cognitive and motivational functions of imagery.

Regarding reliability, the present study achieved acceptable levels of consistency with respect to Cronbach’s alpha (Cronbach, 1951). In fact, results of SIQ:BR were higher in this index than the Finnish (Watt et al., 2006), Spanish (Ruiz and Watt, 2014) and Turkish (Kafkas, 2011) studies, for both individual factors and also the scale as a whole. Cronbach (1951) argues that alpha is a split-half coefficient that shows how consistent the internal structure of responses to a scale are. Thus, it reveals whether it is possible to trust the data, because high values of alpha (above .70) entail an unbiased set of items and sample (Cohen and Swerdlik, 2009). Based on the results obtained by the present study, SIQ:BR is reliable and unbiased.

Finally, the same phenomenon that occurred in Ruiz and Watt (2014) study, also happened in the present paper; significant differences were found between types of sport. Nonetheless, it is important to highlight that effect sizes were small when measured by Cohen’s *f* (ranging between *f* = .11 and *f* = .19), which can lead one to assume that imagery practices do not differ largely across sportsmen. Indeed, Callow et al. (2001) showed that badminton players benefit from MG-M imagery whereas Callow and Waters (2005) employed CS imagery to achieve the same goal with hockey players. Thus, it was expected that different types of imagery might affect different types of sport in different ways. Ruiz and Watt (2014) found that the use of imagery also differed among athletes from diverse sport categories. Thus, imagery use clearly depends on sport category. Given sport category also influenced imagery use in the present study, it can be considered a type of criterion or mixed groups validity, which can be defined as the ability of a test or scale to differentiate groups according to certain criteria (Cohen and Swerdlik, 2009).

Overall, there are some theoretical questions that remain unanswered by this study such as why did MG-A showed lower correlation with other subscales in the Brazilian sample than other studies in the world? And why does frequency of imagery practice differ among types of sports, but not as largely as expected? Regardless, this paper aimed to assess the psychometric properties of the Brazilian-adapted version of SIQ and it seems that this goal was successfully achieved. Content validity was evaluated by judges who agreed with the quality of the adaptation. Factorial and criterion validity were tested and the SIQ:BR presented good results. Also, internal consistency was adequate showing an unbiased and well calibrated measure. Future studies using other statistical approaches are still needed to understand the role of each item in its respective set, however, SIQ:BR is a valid and reliable instrument to evaluate frequency of imagery use among Brazilian-Portuguese speaking athletes.

## Conclusions

Results of studies 1 and 2 provided evidence of content and convergent validity, as well as factorial validity of SIQ:BR that corroborated with previous findings in other countries. Accordingly, group validity demonstrated that types of sport may play a role in frequency of imagery; however, the present research does not allow to reach any final conclusions regarding this aspect. What is possible to ensure is good psychometric properties and evidence of reliability and validity of SIQ:BR.

The main limitation of the present study is that no data about individual differences, practices, and adherence toward imagery was collected or analyzed; thus, it is not entirely possible to suggest that differences of frequency of imagery use between types of sports are exclusively due to categories, then, this issue should be examined in future studies.

## Authors’ information

Dr. Alberto Filgueiras is Adjunct Professor at Universidade do Estado do Rio de Janeiro and coordinates the Laboratório de Neuropsicologia Cognitiva e Esportiva (LaNCE) [Cognitive and Sport Neuropsychology Lab]. He is a former sport psychologist of Team Rio 2016 when he worked with Beach Volleyball Olympic players who participated of Rio Olympics 2016. No standardized instrument measures were available to assess frequency of imagery among athletes, thus psychological evaluation was impaired due to lack of adequate measures.

Dr. Craig R. Hall is Professor at Western University in the School of Kinesiology and developed the Sport Imagery Questionnaire. He is Editor-in-chief of Journal of Imagery Research in Sport and Physical Activity and one of the most recognized experts in imagery in sport around the world.
